# Spatial dimensions of illiteracy in Romania, 1992–2011

**DOI:** 10.3389/fsoc.2022.953870

**Published:** 2022-10-17

**Authors:** Victoria Buza

**Affiliations:** Department of Geography, Faculty of Geography and Geology, “Alexandru Ioan Cuza” University of Iaşi, Iaşi, Romania

**Keywords:** education system, social inequality, social exclusion, literacy level, schooling

## Abstract

Starting with the chapter approaching the educational characteristics of the three census databases carried out in post-communist Romania (1992, 2002, and 2011), broken down at the local administrative unit level, the article aims to analyze from a geographical perspective the phenomenon of illiteracy. In the collective mindset, this notion is primarily associated with poverty, lack of accessibility to education, and/or lack of interest in school. During the three decades covered by this study, influenced by the change of the communist political regime and the economic instability, the complexity of the spatial dimensions of illiteracy is defined by particular demographic, confessional, and ethnic connections which have experienced various dynamics, but following the general tendency of the peripheralization of the phenomenon. Although the Romanian education system adopted certain Western reforms and implemented certain modern strategies, its quasi-obsolete strategies have persisted or have been aggravated in certain well-defined communities, which have been evolving against the downward trend, betraying a severe educational failure, especially in the south of the Romanian Plain, the south of Dobrogea Region, and in Transylvanian Depression. Therefore, in pursuing the implications of the ethnic and religious heritage, as well as the residential area factor, this research is devoted to the study of the main geographical areas where illiteracy is still present and to its relations with the social and economic environment.

## Introduction

In today's Romanian society, the education system is perceived as being in a continuous adaptation and modernization according to European standards, despite the difficulties determined by the sudden transition of the political regime embedded in its substrate, which it, however, gradually manages to overcome. Still, the same education system misses thousands of children every year who fail to be enrolled in school, either because of the unfavorable living conditions of their families or, frequently, because they are total strangers to the idea of “going to school” because of their daily household responsibilities. Thus, there is a real risk that children who initially manifest school absenteeism and dropout will fall victim to the inability to write and read in adulthood. The dimensions of the non-participation in education, seen as a social failure, are exhaustively quantified only in national population censuses and facilitate its approach from certain geographical perspectives, by mapping the statistical data. Along the same line, this topic is widely debated from a plethora of sociological viewpoints, including *school dropout* (Mihalache, 2011; Bonea, 2019), *social and economic inequalities* (Ţoc, 2018; Vasile et al., 2020), and *ethnic segregation* (Hatos, 2011; Arsenie, 2012; Gramaticu, 2012; Anghel, 2019). In the academic literature, the subject of illiteracy is mostly debated in reports of the international organizations (UN, UNESCO), but rarely from a large-scale carto-geographical angle; hence this subject, complex from an ethnic-socio-educational point of view, deserves more attention.

The main objective of the study is to analyze the interactions between space and this social phenomenon, in the context of its natural restriction. In the first part of the study, which focuses on the spatial spread of illiteracy in 1992, an important factor is the age-based breakdown of illiterates, which highlights the majority share of the elderly—children and young people born between the two World Wars, whom the compulsory schooling during the communist period did not assimilate into the system and at the subsequent censuses, they have become fewer and fewer, following the obvious course of the biological exchange of the generations.

However, after the 1990s, the Romanian education system has undergone fundamental transformations due to the change of the political regime from communism to democracy and the transition to the free-market economy, contexts in which education became a permanent topic on the political agenda. Faced with the new reality, the reforms in the education system had to rapidly improve two important aspects: to ensure access to education for the entire school population, especially in the rural areas, which are particularly vulnerable due to the geographical position, school facilities and teaching staff quality, and to adapt the education levels of the population in line with the requirements and competitiveness of the labor market (Muntele et al., 2020). The efficiency of the reform process started in the 90s (mainly focusing on the curriculum reorganization and modernization of the pre-university education) reflects the increase in the number of students enrolled in school and university, simultaneously with the decrease in the number of illiterates in 2002, even though in this period, education received <4% of the GDP (Chiş, 1998).

On the other hand, the educational landscape between 2002 and 2011 censuses is characterized by large-scale changes, strongly influenced by the infusion of neoliberal thinking and a rapid transformation of the Romanian school, with a tendency toward the EU standards. With the integration of (Roma Education Fund., 2007), education has made significant progress, both in terms of increasing public funding and modernizing the school infrastructure, as well as regarding the implementation of programs aimed at reducing the number of people at risk of poverty and social exclusion (Lifelong Learning Program 2007–2013, European Regional Development Fund), which includes dropping out of school or early leaving the education system, as well as the professional integration of non-schooled adults. From the perspective of illiteracy, the connection of the Romanian education system to the European one meant an unprecedented decrease in the number of people who lack basic reading and writing skills, so that, from a statistical point of view, in 2011, the phenomenon was almost eradicated.

However, the illiterate adults in 2002 and 2011 are the former children who faced the social legacy of the political transition period, poverty, segregation, and social inequality, combined with the defining elements of the recipe for school failure: lack of financial resources and parental indifference. Roma, Turkish-Tatar, and Hungarian communities are the most vulnerable regarding school attendance, although even in certain Romanian communities, this phenomenon has not yet been completely eradicated. Additionally, aside from the ethnic inconsistencies, from the point of view of causality, illiteracy manifests at the intersection of *inequalities and social exclusion in schools*: Roma pupils have a poorer lexicon, are more likely to abandon school to get immediate informal income, and have *restrictive gender-related traditions*: many Roma girls leave school at the primary level or are not enrolled in the education system at all, start a family at a very young age, and only 13% of them are employed, compared to 42% of men, the percentages in rural areas being even lower (Arsenie, 2012; Ţoc, 2018).

*School dropout*, another decisive factor for defining illiteracy, essentially reflects family problems, such as economic challenges, migration of adults, the inability of the parents to provide the necessary school supplies, or the total indifference regarding the children's educational performance (Mihalache, 2011; Muntele et al., 2020). Therefore, the geographical distribution of illiteracy is conditioned by the spread of poor areas, large families, low adult education levels, lack of prospects for professional development, and low human capital, where the labor market is dominated by unskilled-job offers (Anghel, 2019), which serve as the perfect elements for the amplification of the cycle of poverty.

The main hypotheses of this study are:

- Eradicating illiteracy is a controllable and plausible process, and its territorial spread having a residual character, manifesting predominantly in rural areas;
- Susceptibility to illiteracy is correlated with belonging to a national minority due to cultural differences and social inequalities.

So, in 2011, there are still 245,523 illiterate people. Where are they? And why?

## Materials and methods

The spatial dimension of this phenomenon, with strong economic and social conjunctures, is influenced by high-level political decisions including geographic components as it outlines certain spatial characteristics and their dynamics in the territory. The main data sources for the number of illiterate persons are provided by national population censuses (published on the website of the National Institute of Statistics), and in this study, those carried out in 1992, 2002, and 2011 were considered. To facilitate the understanding of the geographical areas the study refers to, this article is accompanied by an Supplementary material Annex that includes the raw data of the communes with the highest illiteracy rates and a digital elevation model of Romania with the geographical units mentioned in the text.

The inability to write and read, according to the definition for this situation (Coteanu and Mareş, 2009) is often hidden for various reasons (discomfort, shame, indifference) and may not be visible to the census employee, and, although the censuses aim for the maximum accuracy of the statistical data, sometimes this desiderate can be only partially obtained. In some cases, a dose of skepticism was imposed in relation to the preciseness of the data, especially in the areas predominantly inhabited by ethnic Hungarians (Mureş, Covasna, Harghita, and Satu Mare), where there is an antagonism between the declared ethnicity and the illiteracy rates: there are communes where many Roma people declared themselves to be Hungarians, possible explanations may be the attempt to escape the social stigma they face (Vasile et al., 2020), many of them were assimilated by Hungarian culture and consider that they belong to it, or they are no longer sure about their ethnic affiliation and adapt to the majority's responses (Zamfir and Zamfir, 1993). In these cases, the statistical series are distorted and there is a risk of erroneous conclusions; to strengthen the exactness of the final data and results, the best method was to analyze the correlation of the values of illiteracy with the ethnic and confessional structure, as well as the proximity of communes to urban centers.

Despite these barriers, the statistical analysis and the data mapping methods allow for a sufficiently precise spatial-temporal evolution by observing the dynamics of the territory, the changes of generations, the mentalities and the effects of the political transition, and the socio-educational programs implemented between these periods. Thanks to them, the necessary legislative actions can be determined for targeting the complete eradication of this phenomenon from society and isolate it only in special medical cases.

In the statistical reports, the illiterate population was included in the sections describing the population's general level of education. Hence, the methodological clarifications in the census employees' handbook about the definition of uneducated people (who cannot read and/or write) keep a relatively constant definition for all the censuses, regarding the criteria for establishing the status of “illiterate person.” Therefore, those definitions are presented as follows:

(a) **1992**: For persons born before 1981 (those aged 12 years and over), who have not graduated and are not attending school, it will be written depending on the declaration of these persons, as the case may be: “reads and writes,” “reads only,” and “cannot read or write^1^.”

(b) **2002**: For persons born before 15 September 1991 (those aged 10 years and over), who have not graduated and are not attending an educational institution, the reviewer will write, based on the statement of these persons, as the case may be: “reads and writes,” “reads only,” and “does not know how to write and read.” A person who can read and write a short sentence about everyday life is considered literate, and a person who cannot read and write, or can only read or only write, is considered illiterate. Also, a person who can read and write only numbers and his/her name will be considered illiterate^2^.

(c) **2011**: For persons born before January 2002 (those aged 10 years and over), who have not graduated and are not attending an educational institution, the reviewer will write, based on the declaration of these persons, as the case may be: “reads and writes,” “can only read,” and “cannot read and write^3^.”

In this study, the dissemination of illiteracy is investigated at the LAU^4^ level (communes), and the constant change in the number of these spatial structures required the finding of an intermediate formula that would allow the homogenization and comparison of the data. Consequently, the standardization of communes for all census years is a particularly important methodological aspect, since in 1992, the territory of Romania was organized into 2,946 LAUs, in 2002 into 2,949 (by the establishment of Horia and Costineşti communes in Constanta county and Poienile Izei commune in Maramureş county), and in 2011 into 3,181. To solve the differences, a common map layer was used containing the initial 2,946 communes from 1992 (provided free of charge by www.philcarto.fr and using PhilCarto software) and the raw data of the communes established between 1992 and 2011 were attached manually to the territorial unit from which they were detached. The number of communes to which this study report is 2,943 in 1992 because in three of them, no illiterate persons were registered (Brebu Nou, Caraş-Severin county, Urziceni, Satu Mare county and Secaş, and Timiş county) and in 2011 census, in 41 communes, this phenomenon was not registered. The failure to compare the statistical indicators for the reference year 1992 with 2002 and 2011 due to inconsistency between the methodologically established age limits for those censuses [>12 years (1992), >10 years (2002), and >10 years (2011)] created the necessity of adopting a median data calculation solution. Therefore, the best appropriate method was to compare the illiterate population to the total surveyed population, without separating the age cohorts of the “>12 years” or “>10 years” populations.

Following this procedure, with the same map layer and the databases of the three censuses, the subsequent analyses are based on methods that come from descriptive statistics. Thus, the main statistical indicators that aim to present the spatial dimensions and their dynamics during the 30 years, as far as the reality of the territory and the accuracy of the data allow, are:

- The share of the illiterate population in relation to the total population reviewed;

- The distribution of the standard deviations from the national averages;

- The evolution and the percentage difference of the share/number of illiterates between 2011 and 1992.

The calculation of the proximity of the communes to the nearest urban center was obtained with ArcGIS Pro 10.8 software, in which two data sets were used: the cities and the communes of Romania. Using the Buffer Tool, the Euclidean distances between the urban and the rural settlements were calculated within a radius of 10 (975 communes), 15 (1,791 communes), and 20 km (2,293 communes). About 333 communes remained outside these buffers, most of them being located in remote rural and mountainous areas.

## Results and discussions

**Figure 1:**
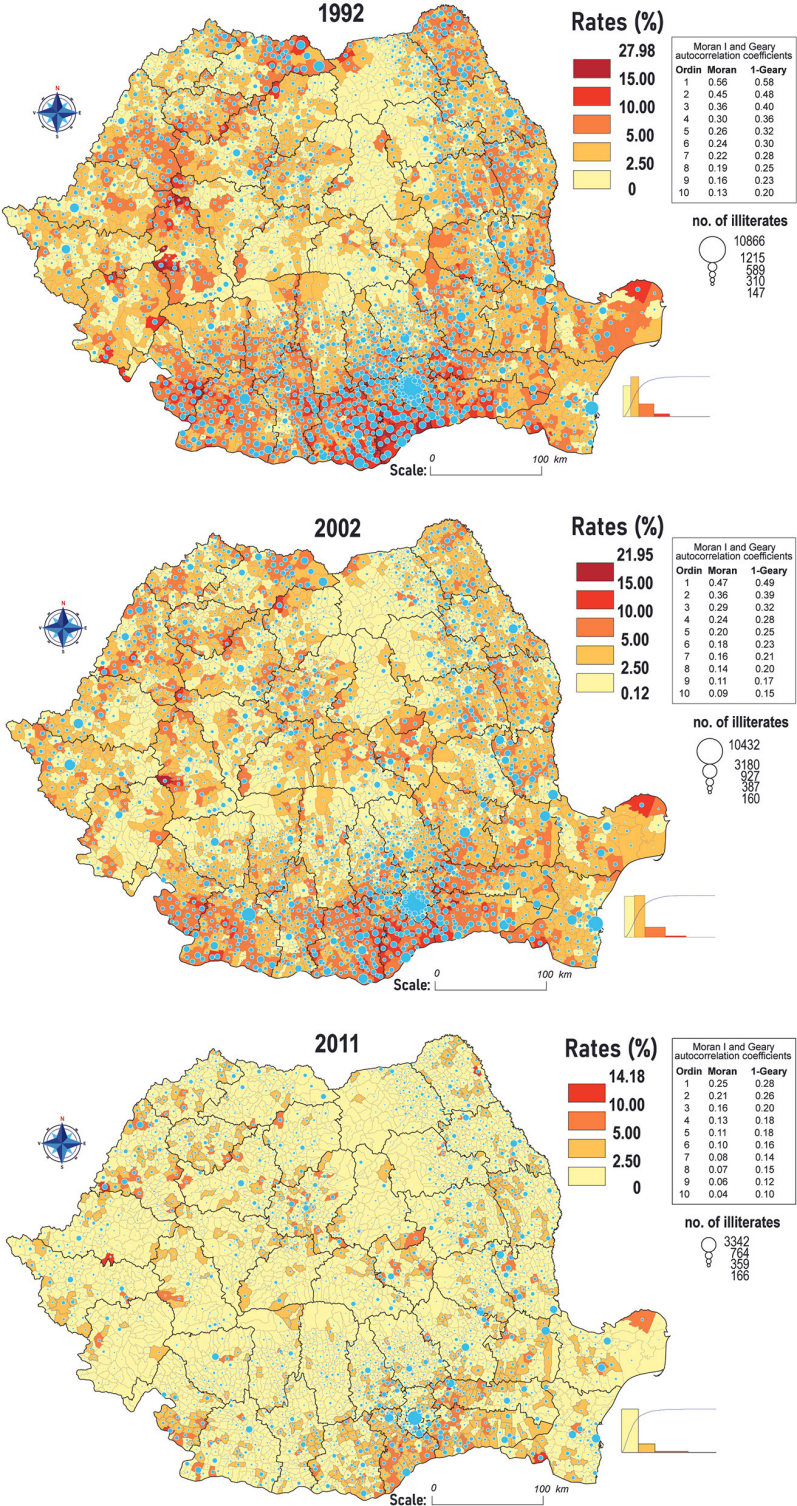
Spatial distribution of illiteracy rates according to the 1992, 2002 and 2011 censuses.

The starting point of this analysis is the mapping of the share of illiterates in the total population over 12 (10) years old, to determine their ratio in each commune, and this calculation required the standardization of color intervals, taking as a reference the 1992 census data when the phenomenon has the largest territorial spreading. The official data used for this indicator were the total population registered and the illiterate population, >12 (>10) years; in this respect, the homogenization of the statistical series allows the comparison of their dynamics, as well as the individualization of some spatial structures. The central areas of the Romanian Plain stand out (Figure 1), especially the polarization area of Bucharest, the central part of the Western Carpathians, and the Maramureş Depression, where the communes with the highest rates of illiterate are concentrated and the maximums recorded in Giurgiu (11.87%) and Teleorman (9.98%) counties form a continuing trend in following censuses.

**Figure 2:**
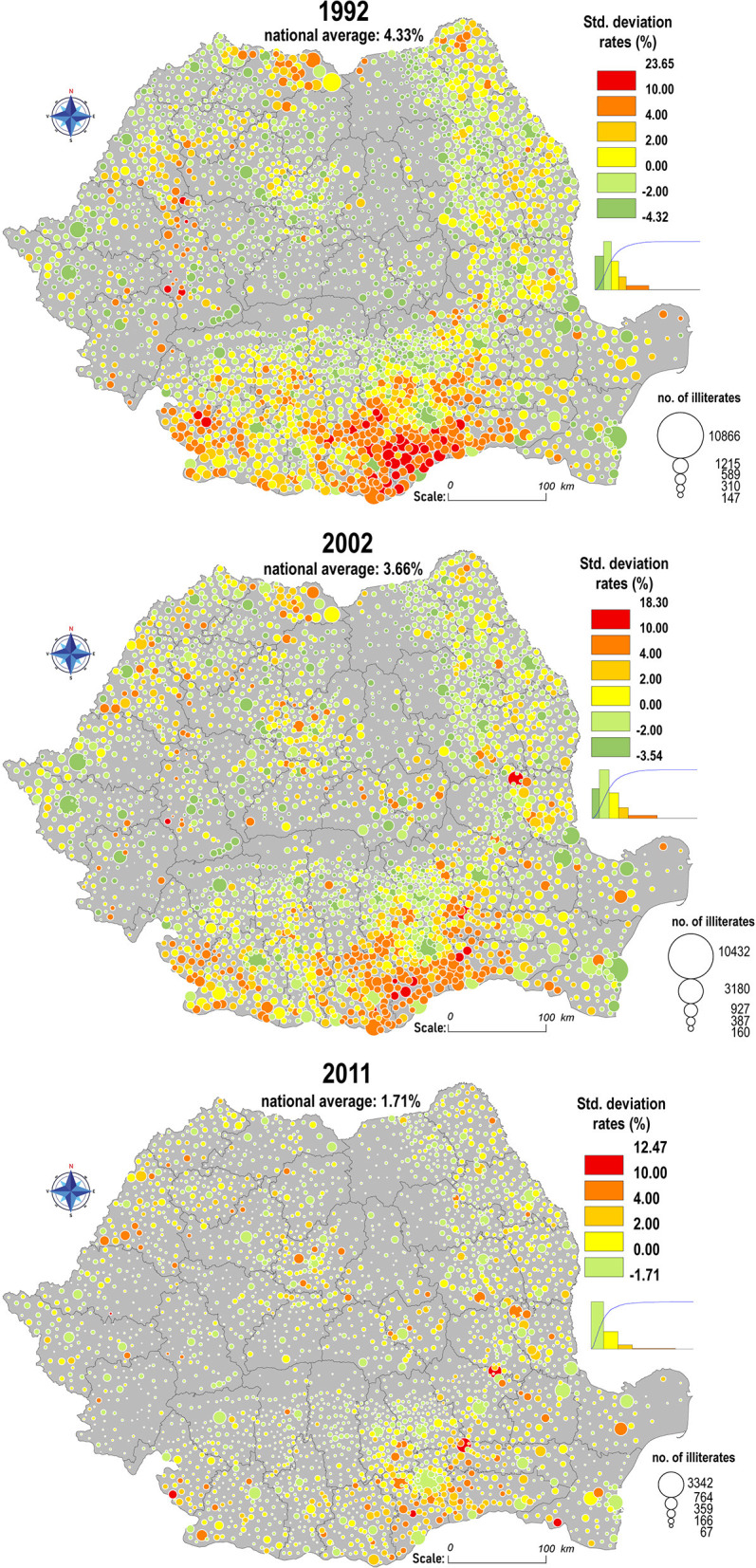
Spatial distribution of the standard deviations rates from the national average of illiterates according to the 1992, 2002 and 2011 censuses.

Obviously, the research of this complex social disability by only calculating the percentages is incomplete, thus, it is also necessary to analyze the standard deviations (Figure 2), which represent the difference between the ratios of each commune (Figure 1) and the national average. Following the methodology of the first cartograms, by the standardization of the values of the color ranges, taking as reference the 1992 census, this indicator highlights the agglutination tendency of the extreme values. The Romanian Plain emphasizes a gradual restraint of the phenomenon, even though Teleorman, Călăraşi, and Giurgiu counties register the largest area of concentration of the positive standard deviations for each of the three censuses. Following the logical course of the natural increase in the general literacy rates for the entire population, the values tend to spread in the Southern Subcarpathian areas, but with reduced intensity.

The dynamics of the evolution of illiteracy in the Transylvanian Depression include areas that have certain particularities in antithesis to the national course, such as Mureş, Covasna, and Harghita counties, where, at a first glance, the phenomenon seems to expand territorially, with the spread of positive deviations in 2002 and 2011. In this case, the correlation of the increase in the general number of illiterate people is linked to the peculiarized ethnic structure of these territories. In addition, Moran I and Geary's spatial autocorrelation coefficients suggest the existence of well-defined spatial structures that coincide with the individualized territories by socioeconomic and ethno-confessional distinctiveness (Târnavelor Plateau, Dobrogea Plateau, and Western Subcarpathians).

In contrast to the mountainous and plain areas, in the historical region of Moldova (the north-eastern part of the country), the dynamics are individualized by the concentration of the low values of the indicators, especially in the Western areas of Suceava, Neamţ, and Bacău counties, while in the Moldavian Plateau, illiteracy has a more prominent presence, but with moderate deviations from the national averages. Taking into account the disadvantages of the central values, it should be noted that the communes of Moldova are represented by lower values of averages and medians compared to the national level in all three censuses: the share of illiterate people in 2011 was 1.61%, (3.76% in 1992) and the median: 1.55%, compared to 1.66%, across the country.

Given the nature of this phenomenon, which is perceived as a failure of the education system and its sudden transitions, which left no room for a smooth adaptation for the new generations, still, its regression across the country is explainable, literacy being a controllable process and has some solvable conjectural causes (extreme poverty, geographical isolation, and low school accessibility). Hereupon, the average national rates decreased from 4.33% in 1992 to 3.66% in 2002 and 1.71% in the 2011 census. In absolute values, according to official statistics, during the 30-years period, the number of illiterates decreases from 591,310 to 245,523 persons.

From a demographic point of view, the temporal dynamics of illiteracy record a natural evolution, in terms of generation replacement: in the 1992 census, the illiterate people of the “>50 years” age group represented 80.06% (83.64% in Moldova, 85.96%, Oltenia, 85.46%, Muntenia, and 67.88% in Transylvania), those of the “25–49 years” age group represent 11.44%, and those 12–24 years old, only 8.49%. The high share of the elderly *and* illiterate is explained by the fact that they were born during both World Wars and the Interwar period, and most of them did not fit into the new literacy reforms initiated by the communist regime in 1948, by Decree no. 175 (Goron, 2018), which stipulated “the elimination of illiteracy” and “the widening and democratization of basic education to include all school-age children as well as the illiterate” (Monitorul Oficial., 1948). At the same time, the success of forced literacy and the centralization of the education system during the communist regime is reflected by the low rates of illiterate people between the ages of 12 and 49 years. Ten years later, the “>50 years” age group of illiterates represents 66.14% of the total, 25–49 represents 18.12%, 10–24 represents 15.73%, and the results from 2002 can be correlated both with the decrease in the number of people born before 1946. An important role was played by the sudden political transitions in society after 1990, also reflected in the education system, when its laws were delayed and many years have functioned without a clear legislation or with few changes, quickly adopted and soon abandoned, depending on the change of the executive (Mitulescu, 2017). This period is also marked by the decentralization of education, reorganization of vocational institutions, and reforms in higher education (Pierson and Odsliv, 2012). Thus, the lack of consistency in the political part of the education system between 1992 and 2002, when the state reorganized itself and the abolition of forced literacy favored a slight increase in illiteracy of young and middle-aged adults, specifically in areas densely populated by Hungarians, in Transylvania Depression and the Western part of Bihor and Satu Mare counties. In Moldova, the phenomenon of illiteracy is manifested mainly in the Moldavian Plain, Tutova Hills, isolated in Bucovina, and is represented by 95% of people over 50 years of age. In the meantime, the southern part of Muntenia region, which concentrates the highest values of illiteracy rates in the whole country, has a particularly accelerated literacy dynamic of the total population, due to the exchange of generations taking into account that it is an aging demographic area and the economic polarization of the capital city, which attracts mainly young people. Thus, in 2002 in Teleorman, Giurgiu, Călăraşi, and Ialomiţa counties, >50-year old illiterate population had the share of 77.34%, compared to 86.91% in 1992 and absolute values from 88,403 to 59,348 individuals. The 2011 census no longer provides a breakdown of the number of illiterates by age group, but even in the absence of these data, the geographical reduction of the phenomenon on a national scale is indubitable.

### Rural vs. urban

**Figure 3:**
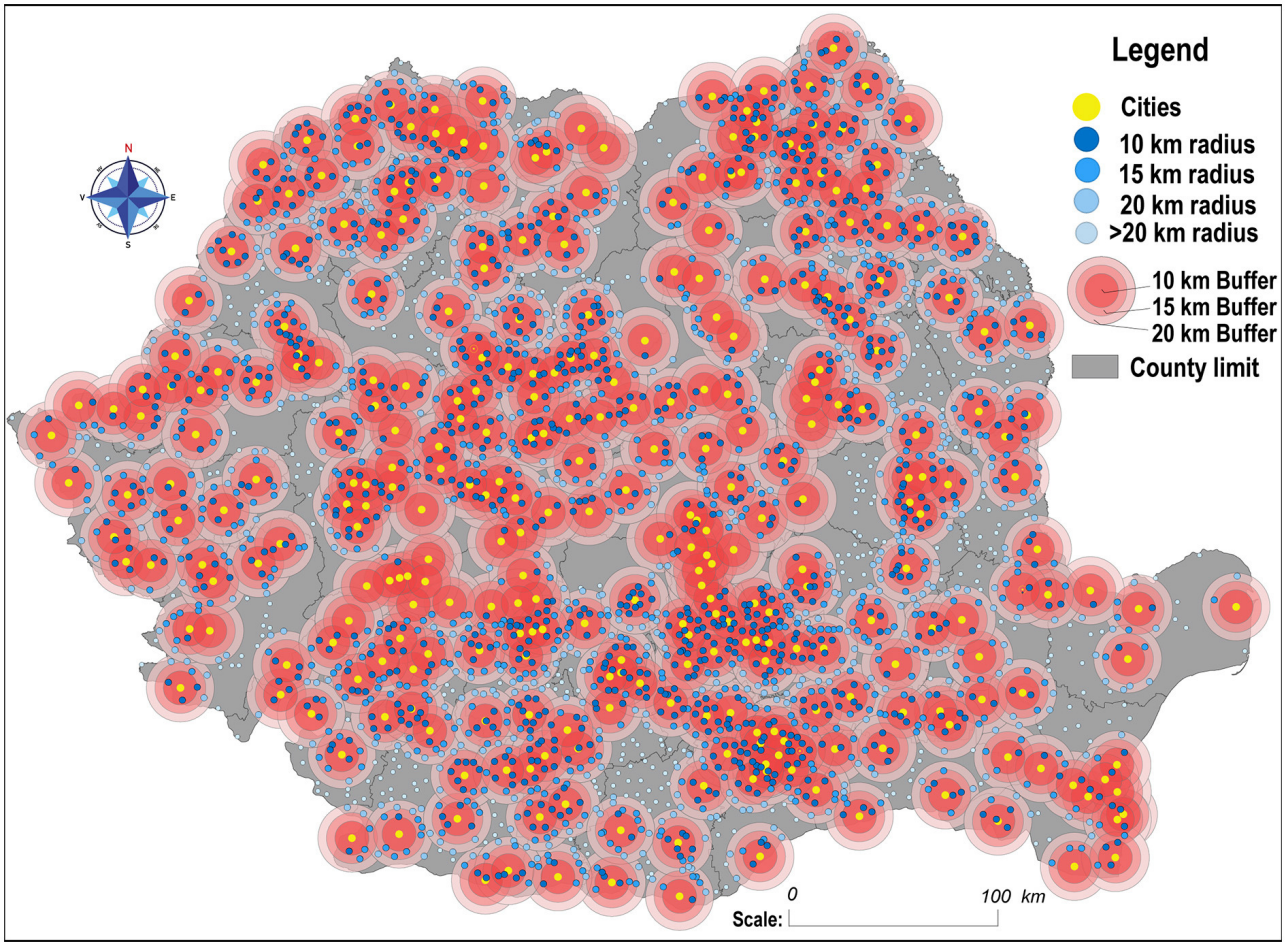
Classification of communes according to proximity to the nearest urban center.

From the perspective of the urban/rural relationship, the analysis of the geographical distribution of illiterate people starts from the assumption of the existence of profound educational inequalities between these two residential areas; the rural environment being more susceptible to the presence and continuity of illiteracy. This situation is caused by a multitude of factors, such as the state's institutional inability to provide equal opportunities to educate all the children, low levels of education of the adult population, lack of jobs, and depopulation of rural areas due to emigration to cities or abroad. Of course, neither the urban nor the rural areas are homogeneous in terms of demographic indicators or economic attractiveness, which is why a further classification of these areas was needed, according to the administrative status (cities and towns) and rural, according to the proximity to the nearest urban center (10, 15, 20, and >20 km; Figure 3).

Illiteracy percentages have an upward trend when increasing the distance between communes and urban areas (Table 1), with steeper edges in 1992, especially between big cities and towns. Moreover, the highest values are distinguished in the remote communities, located more than 20 km away from the nearest urban center and most of them are concentrated in mountainous, sub-mountainous, and deep rural areas (Curvature Subcarpathians, Casimcea Plateau, Godeanu Mountains, Southern Carpathians, and Tutova Hills). Also, the Romanian Plain is differentiated by the agglomerations of communes that are outside the 20-km radius of an urban center in Teleorman (26), Dolj (20), and Călăraşi (10) counties, a region dominated by the demographic and economic influence of Bucharest. The high values of this indicator in their case are explained by the demographic aging, poverty, subsistence agriculture, and lack of financial resources to support the children in school, which leads to their social exclusion, combined with the failure of the state to keep them in the system as long as needed (Pierson and Odsliv, 2012; Benciu and Lădaru, 2014; Cismaru and Corbu, 2019; Petre, 2019).

**Table 1:** Percentage distribution (%) of illiterate people according to the urban and rural environments.

**Settlements/census**	**1992**	**2002**	**2011**
Cities	0.935	1.096	0.604
Towns	2.565	2.430	1.345
Communes in 10 km radius	3.867	3.311	1.576
Communes in 15 km radius	4.209	3.568	1.667
Communes in 20 km radius	4.424	3.730	1.722
Communes outside 20 km radius	5.983	4.778	2.291

### Ethno-confessional conjunctures

The territorial distribution of the illiteracy rates suggests ethnic and confessional correlations. First, the availability of statistical data on the population's ethnicity and literacy level allows the correlation of the shared values of illiterate people, which certifies the hypotheses issued earlier and the advancement of illiteracy research in Romania (Table 2). The strong correlation between the declared ethnicity and the literacy rates is therefore being outlined, despite the fact that the statistical data contain socio-cultural characteristics that are difficult to quantify on a national scale, such as social exclusion, poverty, rigid traditions, inequality, divided families, lack of motivation, or financial struggles. The large percentage differences between the three censuses, with particularly high values for the 2002 Roma and Turkish ethnics and the similarities between the 1992 and 2011 censuses, are conditioned by several interspersed and subjective factors such as a non-exhaustive collection of data of some census sectors, registration of other ethnicities than the *de facto* one, reluctance to answer the questions honestly, progress in the literacy of vulnerable minorities, and so on.

**Table 2:** Percentage distribution (%) of illiterate people by ethnicity in 1992, 2002, and 2011.

**Ethnicity/census**	**1992**	**2002**	**2011**
National_avg	2.59	2.62	1.36
Romanians_avg	2.48	2.14	1.01
Hungarians_avg	0.89	1.35	0.81
Roma_avg	14.24	25.62	14.13
Ukranians_avg	6.73	5.70	2.05
Lipovans_avg	5.95	4.67	2.24
Turkish_avg	12.85	23.74	11.12
Other_avg	1.19	1.34	0.63

Taking into account the major changes that develop over the 30-year period, in addition to the peripheralization of this social aspect, the mountainous areas are distinguished by the lowest registered values and, besides that, they still greatly reduced their diffusion range in 2011. However, the phenomenon is gaining intensity rather locally in Mureş county in the communes with a diversified ethnic structure, consisting mostly of Hungarians, Roma, and Romanians (Eremitu, Brâncoveneşti, Band, and Mica), a similar situation in Covasna, where the communes in which the dominant ethnicities are Hungarians and Roma, there is an involution of the literacy process (Brădut, Ghelinta, and Ojdula), similarly in Bihor (Chiumeghiu, Lăzăreni, and Abrămut). For example, in the communes of the Mureş Plain, Târnăvenilor Hills, where Roma people are the majority, there are large shares of the illiterate population.

The best way to find out the causes of these peculiarities was to directly contact the local authorities; they preferred to provide information under the protection of anonymity regarding the inconsistencies between the official census data and the real situation in the isolated villages both geographically and socio-ethnically. The local social assistance department and teachers from Mureş, Bihor, and Covasna confirmed that the data on the ethnic structure do not precisely reflect the social reality, as the majority of the Roma population declared themselves Hungarian (their children attend schools teaching in Hungarian, in some villages being the only available school), instead, in ethnic Hungarian families, illiteracy is almost non-existent. The increase in the values of this indicator can also be attributed to the high fertility rate, specific to this Roma ethnicity, as a result of the traditional lifestyle (4–5 children/woman) (Preda, 2002), contrary to the other communities (1–2 children/woman). In addition, women in Roma communities are much more likely to be illiterate or achieve a lower school level than men (according to the 1992 census data, 35.2% of Roma women were illiterate compared to 18.6% of men), and most of those who attend school, however, stop at the 8th grade [about 4% follow the high school and higher education is rarely encountered; (Zamfir and Zamfir, 1993)]. The main reasons are related to cultural values, marriages, and early taking on the roles of housewife and mother (Bădescu et al., 2007; Arsenie, 2012). Furthermore, the ethnic segregation and marginalization of Roma minorities intensified after the change of the political regime and the valorization of schooling is questioned by adults, based on the historical perspective of the persistent social inequalities (Pierson and Odsliv, 2012), and because of the lack of successful role models thanks to higher education. Moreover, the barriers of society's negative attitude toward Roma children, worsened by the low level of education of their parents and their seasonal emigration, create a vicious circle of their academic exclusion, causing low enrolment rates in primary school (Advancing Education of Roma in Romania, 2007).

The versatility of the statistical data is mainly caused by social stigmatization, the “romanization” of a consistent number of Roma people (Gábor and Rughiniş, 2008). This phenomenon, increasingly accentuated in the past two decades, translates into the non-identification of Roma people with their ethnicity and the non-recognition of their ethnic affiliation and their absorption by the Romanian/Hungarian communities (Preda, 2002). To dilute the ethnic confusion between Roma and Hungarians caused by the subjectivity in stating one's ethnicity, the communes were divided according to the proportions of the declared ethnicity.

In 2011, 381 communes had over 10% Hungarian population, fewer compared with the previous censuses: 402 communes in 2002 and 417 in 1992, and the share of the illiterate population increased from 7.8 to 13.4%. By comparison, in 2011, in the 167 communes, where more than 50% of the Hungarian population was surveyed (177 communes in 1992), there was a decrease in the illiterate population compared to 1992: from 2.23 to 1.65%. Thus, it is obvious that the increase in the share of illiterate people is taking place in communes with diversified ethnic structures, especially where there are declared Hungarians and Roma. Additionally, upon closer analysis, in 2011, there are 417 communes where Roma make up >5% and Hungarians <1%, and in these spatial units, the share of illiterates increased by 5.6% between 1992 and 2011. To certify the idea that the dynamics of these spatial structures are shaped mainly by the Roma communities, vulnerable to the phenomenon of illiteracy, the next step was the separation of the communes with over 20%, 10% Roma (374 in 2011, 177 in 1992), and those with over 5% (732 communes in 2011, 404 in 1992), showing the increase of 8.1% (>10%) and 10.4% for the second range (Table 3). A similar situation is found in the localities where, in 2011, the share of illiterate people exceeds 10% (21, compared to 72 in 2002 and 280 in 1992): Armăşeşti, (Ialomiţa), Siştarovăt, (Arad), Stoeneşti, and Găujani (Giurgiu) communes, where over 98.5% of the inhabitants declared themselves to be Romanians.

**Table 3:** Distribution of illiterate percentages and deviations in communes within 5–>15% Roma ethnics.

**% Illiterate/census**	**1992**		**2002**		**2011**	
	**% Percentage**	**% Deviation**	**% Percentage**	**% Deviation**	**% Percentage**	**% Deviation**
>15% Roma ethnics	5.33%	1.00%	6.34%	2.68%	3.52%	1.80%
>10% Roma ethnics	5.17%	0.84%	5.80%	2.14%	3.26%	1.55%
>5% Roma ethnics	4.96%	0.63%	4.90%	1.24%	2.65%	0.94%

In the same time, the areas of Dobrogea Plateau present distinct features in terms of literacy rates, associated with the territorial spread of the Turkish-Tatar population, mostly in Constanta and isolated in Tulcea. In this region, illiteracy reaches some of the highest values during all censuses, strongly influenced by ethnic factors. Hence, in the communes where these ethnic minorities form the majority of the total population, the illiteracy values are elevated and the decrease in the number of ethnic Turkish-Tatars is proportional to the decrease in the percentages and standard deviations (Table 4). For example, in the LAUs with >15% Turkish-Tatar people, this indicator maintains the highest values, while the second category (>10%), which includes ethnically diverse cities like Constanşa, Mangalia, Eforie, Hârşova, and Ovidiu, leans the balance toward higher levels of education.

**Table 4:** Distribution of illiterate percentages and deviations in communes with 5–>15% Turkish-Tatars ethnics.

**% Illiterate/census**	**1992**		**2002**		**2011**	
	**% Percentage**	**% Deviation**	**% Percentage**	**% Deviation**	**% Percentage**	**% Deviation**
>15% Turkish-Tatars ethnics	7.25%	2.92%	5.49%	1.83%	4.61%	2.90%
>10% Turkish-Tatars ethnics	6.72%	2.39%	5.22%	1.56%	4.18%	2.47%
>5% Turkish-Tatars ethnics	5.15%	0.82%	4.10%	0.44%	2.76%	1.05%

To detail the analysis of the correlation between illiteracy and the declared religion, the Pearson correlation and determination coefficient between the share of the illiterates for each confession or group of confessions were used (Tables 5, 6). Consequently, % *Orthodox* include both Orthodox and Old Rite Orthodox, % *Catholics* include Roman Catholics and Greek Catholics, % *New Protestants*: Baptists, Adventists, Pentecostals, and Evangelicals, % *Protestants*: Reformed, Lutherans, and Unitarians; and persons who declared to be atheists or without religion were considered separately.

**Table 5:** Pearson correlation coefficient between illiteracy ratio and religion (r).

**Religion/census**	**% Illiterate 1992**	**% Illiterate 2002**	**% Illiterate 2011**
% Orthodox	**0.294**	**0.149**	0.025
% Catholic	**−0.217**	−0.100	−0.047
% New protestants	−0.080	−0.066	0.112
% Protestants	**−0.208**	−0.049	0.004
% Muslims	0.014	0.047	**0.140**
% Atheists	−0.114	−0.093	−0.045
% Without religion	−0.089	−0.009	0.013

**Table 6:** Determination coefficient for Pearson correlation between the illiteracy ratio and religion (P-value).

**Religion/census**	**% Illiterate 1992**	**% Illiterate 2002**	**% Illiterate 2011**
% Orthodox	*0.086*	*0.022*	**0.001**
% Catholics	*0.047*	**0.010**	**0.002**
% New protestants	**0.006**	**0.004**	0.012
% Protestants	*0.043*	**0.002**	0.000
% Muslims	0.000	**0.002**	0.020
% Atheists	0.013	**0.009**	**0.002**
% Without religion	**0.008**	0.000	0.000

The highest coefficients are obviously for the Orthodox confession (0.294, 0.149), since the vast majority of the population declared their affiliation to it, therefore, due to collinearity, it can be considered a redundant value. In addition, in the 1992 census, two negative correlations (-0.217 and -0.208) of the Catholic and Protestant confessions (predominant in the west of the country) stand out, highlighting the long tradition of compulsory schooling that these communities had, but this trend fades in the following censuses. Furthermore, in order to determine how strong the link between confession and the vulnerability of being illiterate is, the Pearson determination coefficient was calculated (Table 6). Starting from the fact that in this statistical measurement 0 indicates no correlation and 1 represents a perfect match, most of the values are very close to 0 (in bold), which means an almost non-existent connection or a very weak dependence (in italics). Thus, religious affiliation can partially explain the spread of illiteracy only in isolated cases. For example, the correlation between the confession and the susceptibility of the Muslim community toward illiteracy (0.140 in 2011) is manifested in the Southern part of Constanţa, especially in Dobromir, Castelu, and Băneasa, which recorded standard deviations of >5% (Dobromir, 12.97%). Similar to Mureş and Bihor, local authorities confirmed the magnitude of the phenomenon, especially among young adults. Nevertheless, for Turkish-Tatars, the situation is worsened primarily by the lack of the parent's involvement during the school years and their disregard for the importance of school and getting an education. Additionally, the language barriers make the literacy process difficult (the schools' teaching language is Romanian, and Turkish is predominantly spoken outside the school gates), so many children end up leaving school, especially girls. At the same time, according to local authorities from Bihor, Mureş, and Constanţa, the implementation of the Ministry of Education's “Second Chance” national program faces a low interest from the targeted population and it does not play a significant role in the mass schooling of adults who have not graduated the primary level.

It is important to note that this situation is not found in urban centers with several thousand Turkish-Tatars such as Constanţa (18.751), Medgidia (7.997), and Mangalia (3.242). In the urban environment, due to its high accessibility to educational resources, the modern and accelerated lifestyle manages to literate almost all the population, including ethno-confessional communities which in rural areas would be less willing to go to school. After all, the dynamics of the spatial structures of illiteracy in southern Dobrogea are determined by the combination of both linguistic and ethnic peculiarities and conservative lifestyle (Abdula-Nazare, 2021).

On the other hand, the correlation analysis shows that religious affiliation is not a strong determining factor for the spatial distribution of illiteracy, unless ethnicity is also taken into account. Of course, when applying similar analysis at regional or local level, the values are higher and the correlations stronger; for example, Turkish-Tatar Muslims in Constanţa or New Protestants in Western Transylvania. Despite this, it is important to mention that the entire zonal religious distinctiveness is attenuated when zooming out to the national scale and the links of the illiteracy distribution and its dynamics are nuanced by ethnic, economic, and social components rather than by religious factors. The *P*-value test (Table 6) analyzes the preciseness of the hypothesis: the correlations of *% Orthodox* (1992, 2002), *% Catholics* (1992), and *% Protestants* (1992) have 5% significance, and the values of <0.01 are very significant as they validate the factuality of the analysis by 99%.

### Illiteracy evolution: Unfavorable circumstances or lost cases?

**Figure 4:**
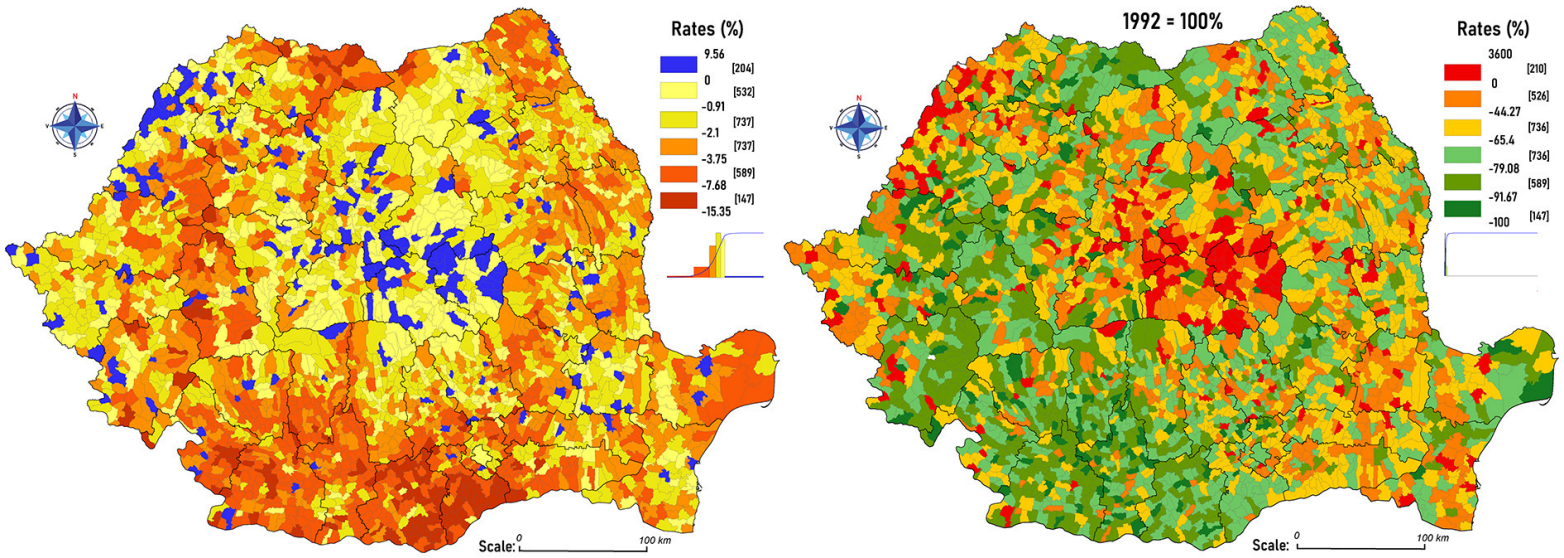
**(A)** The percentage evolution in the share of illiterates between 2011 and 1992 and **(B)** The percentage differences in the number of illiterates between 2011 and 1992.

To deepen this discussion, the cartographic representation of the percentage evolution of the share of illiterates between 2011 and 1992 was used (Figure 4A) and the calculation of the percentage difference of their number was done considering 1992 as a reference year (Figure 4B). The formulas used are:

For the percentage difference in the share of illiterates between 2011 and 1992:


$$\begin{array}{l} \frac{Illit_{2011}\;\cdot \;100\%}{Pop_{2011}}\;-\;\frac{Illit_{1992}\;\cdot 100\%}{Pop_{1992}} \end{array}$$

and the percentage difference of the illiterates' number between 1992 and 2011:


$$\begin{array}{l} \frac{Illit_{2011}-\;Illit_{1992}}{Pop_{1992}}\;\cdot 100\% \end{array}$$

These calculations reinforce the accuracy of the available statistical data and confirm that illiteracy has evolved from a structural phenomenon to a conjunctural one. The residual character is highlighted only in certain rural areas with the special ethnic/social/confessional particularities mentioned earlier. The first formula reflects the natural contraction of the illiterate individuals from the total population >10 years [>12 years (1992)] in most of the country, excepting 177 of them (Figure 4A). Their dissemination may seem spontaneous but the spatial structures with positive values are mostly agglutinated in the center of the Transylvanian Depression, at the intersection of Harghita, Covasna, Mureş, and Braşov counties, where there is a high ethnic diversity: there is an aggregated area with communes that are distinguished by their ethnic structure with over 57% Hungarian, 11% Roma, and 27% Romanians. A similar situation is found in the Western part of Bihor and Satu Mare counties and isolated in Sălaj. Excluding these cases, the territorial shrinking of this social handicap is undeniable: major percentage differences are found in the Romanian Plain: Giurgiu (−7.61%), Teleorman (−7.35%), Olt (−4.65%), and in the Western Carpathians area: Hunedoara (−3.99%). The evolutions are correlated with the accentuated demographic aging of these territories.

The analysis from Figure 4B highlights the differences between the total number of illiterates between 2011 and 1992, negative values are noted almost throughout the country, the top counties being Olt (−77.95%), Teleorman (−77.57%), and Dolj (−75.42%), and Covasna (+38.64%) being the only county with positive values. This calculation outlines problem areas with precise territorial boundaries: the most extensive being in the Transylvanian Depression, and isolated in other counties, where these communities face both economic and motivational challenges, are still skeptical regarding the long-term benefits of the education and prefer to work during the school years for a rapid income. In addition, this is a sensitive topic to be discussed as some issues, such as normal school attendance and the necessary financial investments, fall into dissonance with certain traditions and the daily responsibilities of the children in the household (Mihalache, 2011).

## Discussion

The research focused on the accuracy of the official census data, taking into account the underlying subjectivity due to the sensitive and personal nature of the questions related to educational characteristics. The study is still an exploratory one, with an analytical character, aimed to explore the hypotheses that explain the structures of spatial illiteracy. The geographical analysis of their dynamics is essential to investigate the natural limitation of this phenomenon that can be used to improve future educational policies, focusing particularly on those areas where illiteracy persists. At the same time, the official data, being confronted with the information from the local authorities, reinforce the fact that it should be used rather as a guide mark, since in many cases the situation may be different and usually worse. Undoubtedly, the feeling of shame often combined with indifference and lack of future visions about the importance of education plays a decisive role in the correctness of the answers of the people who do not know how to write and read, and consequently, the true spatial distribution of illiteracy in Romania can only be estimated. The first hypothesis launched, regarding the controllable and residual nature of the phenomenon, has been partially proven: the analysis of the data confirms the inextricable path of the increase of literacy levels due to generational changes and better public education financing, especially after Romania's integration into the EU, and general growth of lifelong education. In the 1992 census, the national registered average of illiteracy was 4.33%, spread throughout the country, especially in the Southern part of the country, where there are areas with over 20% of illiterate people. The residual character is manifested in the subsequent censuses, an important psychological factor that supported this transition in Romanian society was the idea that having an education is the best way to ensure a better quality of life and optimistic perceptions for the future. In poor areas, school is often seen as the best way out of poverty. The calculations of the variations of illiterate shares in relation to the distance from the nearest urban center indicate that urbanization is a decisive factor in reducing illiteracy, but even in the urban environment, it cannot be eliminated definitively. In the meantime, there are areas where ethnic minorities face poverty, social inequalities, and exclusion; in these communities, there is an increased risk of illiteracy among the new generations and erasing it may take more time. The statistical analyses of the positive correlations between the ethnic belonging and the vulnerability of being illiterate validate the second hypothesis. Either way, the phenomenon will be gradually shrinking but could be completely eradicated through the application of accelerated local literacy programs, individualized according to the particularities of each community.

The results of this geographical analysis, although they highlight the problem areas with heterogeneous characteristics, should be seen as a starting point for future scientific research. In this context, the collaboration between the local and central government is the key] factor responsible for establishing and implementing effective schooling strategies to ensure that no child is left behind.

## Ethics statement

The studies involving human participants were reviewed and approved by University Alexandru Ioan Cuza, Doctoral School of Geosciences. Written informed consent for participation was not required for this study in accordance with the national legislation and the institutional requirements.

## Author contributions

The author confirms being the sole contributor of this work and has approved it for publication.

## Funding

I thank the Doctoral School of Geosciences for its contribution to this research. The logistical and financial support included the transportation and accommodation to the areas with the highest rates of illiteracy, which undoubtedly contributed to establishing clear perspectives on the causes and evolution of this phenomenon.

## Conflict of interest

The author declares that the research was conducted in the absence of any commercial or financial relationships that could be construed as a potential conflict of interest.

## Publisher's note

All claims expressed in this article are solely those of the authors and do not necessarily represent those of their affiliated organizations, or those of the publisher, the editors and the reviewers. Any product that may be evaluated in this article, or claim that may be made by its manufacturer, is not guaranteed or endorsed by the publisher.

## Footnotes

1 Comisia Centrală pentru Recensământ. (1991).

2 Comisia Centrală pentru Recensământ. (2002).

3 Institutul Naţional de Statistică. (2011).

4 Local Administrative Unit = commune.
