# Supplementary material for: Spatial dimensions of illiteracy in Romania, 1992–2011
Source: Front Sociol. 2022 Oct 17;7:953870. doi: 10.3389/fsoc.2022.953870 (PMC9618730; doi:10.3389/fsoc.2022.953870)

**Annex**

Table 1 Top 15 communes (LAUs) with the highest illiteracy precentage in 1992, 2002, 2011 censuses

Figure 1 Main relief units and counties of Romania

| **Commune** | **County** | **%1992** |  | **Commune** | **County** | **%2002** |  | **Commune** | **County** | **%2011** |
| --- | --- | --- | --- | --- | --- | --- | --- | --- | --- | --- |
| Stoenești | Giurgiu | 27.98 |  | Stoenești | Giurgiu | 21.95 |  | Slobozia Bradului | Vrancea | 14.19 |
| Toporu | Giurgiu | 24.05 |  | Lunca Cernii de Jos | Hunedoara | 16.93 |  | Stoenești | Giurgiu | 13.84 |
| Lunca Cernii de Jos | Hunedoara | 20.24 |  | Toporu | Giurgiu | 16.07 |  | Șiștarovăț | Arad | 13.62 |
| Găujani | Giurgiu | 19.07 |  | Sărulești | Călărași | 15.39 |  | Armășești | Ialomița | 13.00 |
| Brânceni | Teleorman | 18.47 |  | Armășești | Ialomița | 14.30 |  | Gruia | Mehedinți | 12.80 |
| Pădina | Mehedinți | 18.47 |  | Bujoreni | Teleorman | 14.21 |  | Dobromir | Constanța | 12.30 |
| Răsmirești | Teleorman | 17.68 |  | Brânceni | Teleorman | 14.09 |  | Vidra | Ilfov | 10.95 |
| Gratia | Teleorman | 17.65 |  | Izvoarele | Giurgiu | 14.00 |  | Ciumeghiu | Bihor | 10.81 |
| Izvoarele | Giurgiu | 17.51 |  | Brăhășești | Galați | 13.82 |  | Bolintin-Vale | Giurgiu | 10.57 |
| Gogoșari | Giurgiu | 17.50 |  | Sohatu | Călărași | 13.71 |  | Conțești | Dâmbovița | 10.11 |
| Bătrâna | Hunedoara | 17.42 |  | Schitu | Giurgiu | 13.49 |  | Mihălășeni | Botoșani | 10.00 |
| Sohatu | Călărași | 17.27 |  | Răsmirești | Teleorman | 13.37 |  | Brăhășești | Galați | 9.78 |
| Bragadiru | Teleorman | 17.05 |  | Orbeasca | Teleorman | 13.25 |  | Sărulești | Călărași | 9.77 |
| Răchitova | Hunedoara | 16.80 |  | Vărăști | Giurgiu | 13.19 |  | Toporu | Giurgiu | 9.52 |
| Văleni | Olt | 16.50 |  | Mihai Bravu | Giurgiu | 12.91 |  | Vânjuleț | Mehedinți | 9.48 |
| *National average* | | *4.33* |  | *National average* | | *3.66* |  | *National average* | | *1.71* |


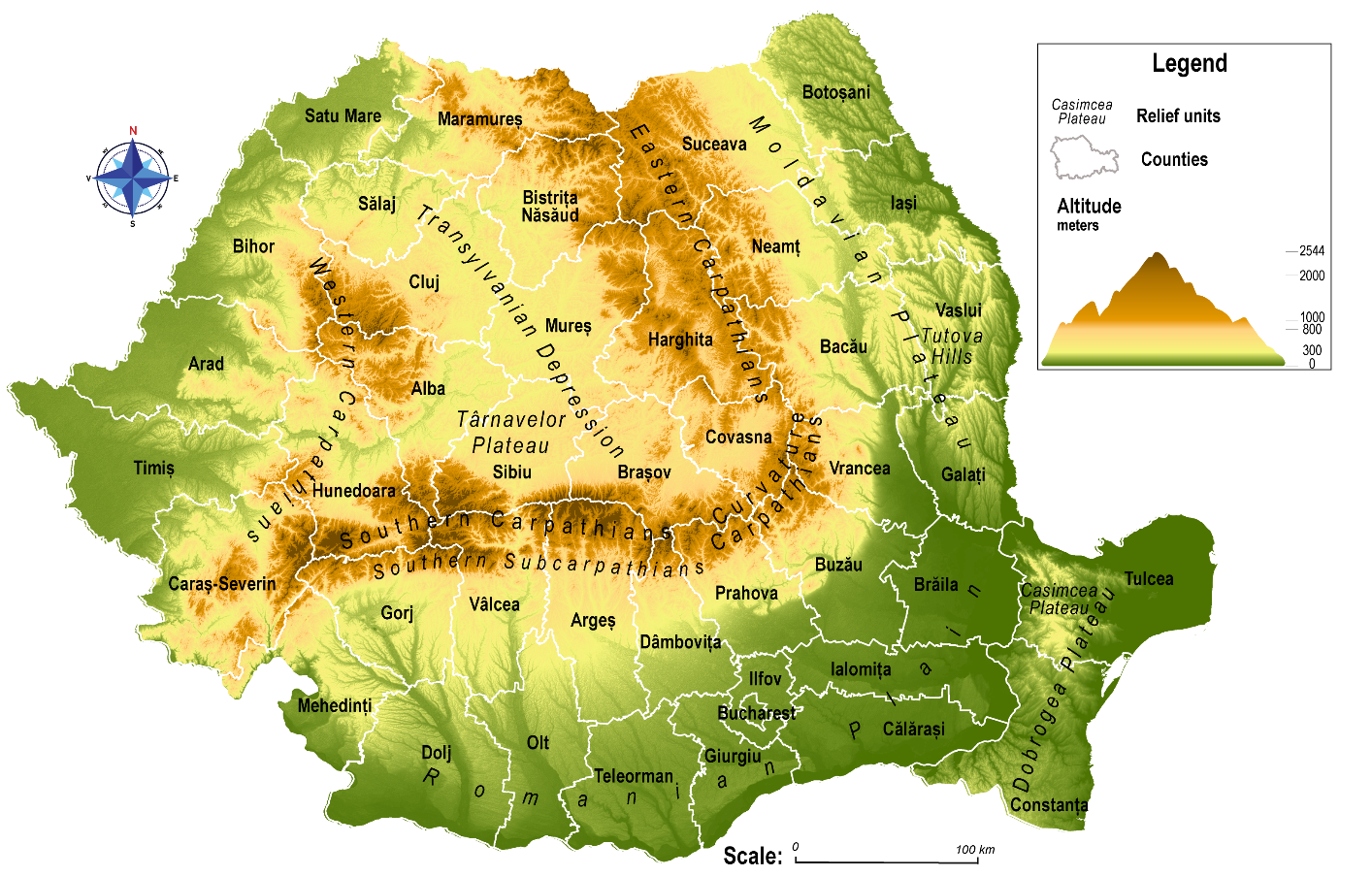

Supplement: Supplementary file 1 [file Table_1.docx]
